# Healthcare information systems: the cognitive challenge

**DOI:** 10.1186/s12911-018-0584-z

**Published:** 2018-01-11

**Authors:** Gavan Lintern, Al Motavalli

**Affiliations:** 1 Monash University Accident Research Centre, Building 70, Monash, VIC 3800 Australia; 2 Department of Anaesthesia, The Northern Hospital, 185 Cooper St, Epping, VIC 3076 Australia

**Keywords:** Electronic healthcare records, Cognitive engineering, Cognitive design, Decision-centered design

## Abstract

**Background:**

Healthcare work is, to a considerable extent, cognitive. Subsequently, the analysis and the design of supporting technology must be sensitive to the cognitive and adaptive demands of the work and to the cognitive strategies employed by healthcare practitioners. Despite the vital role that cognition plays in healthcare work, current technocentric design approaches for healthcare technology do not account for it, failing to observe it during analysis and failing to develop support for it during design.

**Main body:**

By review and analysis of case studies, we show that healthcare systems developed without input from cognitive analysis and cognitive design fail to take account of important healthcare work processes and workflows. In contrast, systems developed with a cognitively-focused design strategy demonstrate how it is possible to introduce technology that supports and enhances the work strategies of those engaged in patient care.

**Conclusion:**

Significant problems emerge when technological support systems are developed without any serious and comprehensive attempt to understand the cognitive capabilities and skills deployed by those involved in patient care. In contrast, significant benefits accrue from taking full account of those cognitive capabilities and skills. Subsequently, the design and development of supporting technology must be sensitive to the cognitive demands of the work and the cognitive strategies employed by healthcare practitioners.

## Background

Healthcare is an information rich environment [1]. It is complex, involving diverse, interdependent, knowledge-intensive disciplines, and is dynamic, involving knowledge that is being constantly revised and extended [2, 3]. This information environment extends beyond clinical care to include management, funding, and policy [1, 4] and beyond the hospital to include home care and primary care [3].

Policymakers have promoted the development and use of computerized information systems under the assumption that they will improve quality, efficiency, and safety of healthcare [5, 6]. Indeed, at first glance, computerization of such an information environment would seem to offer enormous organizational, safety, performance, and economic benefits. There is, however, a troubling discrepancy between the attitudes of different professional groups to healthcare information systems, with clinicians being less satisfied than information technology professionals [7] and less satisfied than health plan executives [8]. Notably, healthcare professionals are often positive about the introduction of innovative technology, only to become disenchanted when they discover that it disrupts their workflow [9].

The dissatisfaction of clinicians with healthcare information systems suggests that many efforts at computerizing healthcare information have not fulfilled their potential, at least in the realm of patient care. Greenhalgh et al. [4] found no evidence that computerized information systems produce the anticipated healthcare benefits, while Greenhalgh and Stones [5] question the assumption of policymakers that such systems will improve quality, efficiency and safety. Furthermore, clinicians have experienced them as disruptive and inefficient [9, 10]. More recently, usage rates have been reported at above 90%, but dissatisfaction with the impact of healthcare information systems on workflow and patient throughput remains high [11].

The current approach to development and design of healthcare information systems is guided by a rational, technocratic world view [2, 4] that substitutes designer judgment for clinician judgment [10]. The design solutions that emerge from this rational model rely heavily on overly-simplified decision-support rules that disrupt clinical workflows, and on inflexible templates and standardized protocols that do not accommodate the complex and dynamic challenges posed by diverse health issues [4, 10]. The ensuing systems fail to account for the demands of collaborative clinical work in which healthcare professionals must contextualize and prioritize knowledge to cope with multiple workflow possibilities and non-routine conditions [2]. This gulf between the reality of clinical work and how it is rationalized for information-technology design leads to development of systems that induce error and do not properly support the work as intended [2, 4, 10].

As reported by Challenger and her colleagues, the experience of the UK National Health Service in implementing its Care Records Service is illustrative [12]. The Care Records Service is an electronic healthcare record developed to manage medical records for all patients in the system. The overarching vision for the Care Records Service evolved from an unquestioning belief that healthcare needs to take advantage of new technological developments [12]. Better patient care would emerge naturally from deployment of automation technologies that would perform specific healthcare tasks and from a suite of information technologies that would allow healthcare practitioners ready access to the most up-to-date patient information.

Policymakers assumed that transfer of tasking to automation and ready access to current patient information would improve clinical decisions via more efficient consultations, and thereby promote more effective and more economical care while reducing the risk of medication errors [12]. Nevertheless, despite the many apparent benefits of computerization, there was widespread evidence of issues relating to the cognitive work; a lack of compatibility of system configurations with clinical practice, incomplete and inaccurate information, a restrictive data entry strategy, and an electronic-notes function that increased the cognitive work associated with taking a patient history, to name just a few [12]. There was little evidence of the anticipated benefits.

### Cognitive design

In large part, the responsibility for the design, development, implementation and modification of healthcare information systems is left to software engineers and information technologists whose appreciation of the scope and complexity of healthcare work-as-done is necessarily limited [4, 10]. Experienced administrators and clinicians who select from commercially available systems typically have little to no expertise in device evaluation [13]. Opportunities for healthcare professionals to influence the design are fragmented at best and typically amount to little more than an incomplete list of functional requirements as developed by a small, non-representative, albeit knowledgeable group of healthcare specialists [10]. Nothing about this process approximates a design strategy that could lead to a coherent, robust and effective information system to support the diverse and complex demands of healthcare work.

Although healthcare information systems are focused on the cognitive dimensions of the work [10], they reveal no sensitivity to an insight from the field of situated cognition; that workers converge naturally on robust and powerful ways of doing work that differ markedly from the formal strategies that emerge from the technocratic world view [14, 15]. Nor do they reveal any sensitivity to the insight from Naturalistic Decision Making that experts in the field, who are making critical decisions under high workload and time pressure, do not follow a rational strategy of options analysis but rather, recognize and act spontaneously, choosing a different strategy only if the first proves to be unsatisfactory [16].

Absent sensitivity to these insights, healthcare information systems will, at best, fail to support the informal but powerful strategies used in patient care. At worst, healthcare information systems will disrupt and block these strategies, thereby inducing new and unanticipated systems errors and forcing those involved in patient care into work patterns and work arounds that are fragile, error inducing, and labor intensive.

There has been a recent call for healthcare to embrace the principles of high-reliability organizing [17, 18]. Despite undertaking complex and risky work, high-reliability organizations achieve exemplary levels of safety [19]. They do so in part by remaining mindful of the subtle and complex details of operational work [20, 21]. In that respect, procedural constraints embedded in technology should not interfere with operational work and institutional demands should not be prioritized over operational demands [20]. Technology is a crucial element of today’s high-reliability organizations [19], but those who design computerized information systems for healthcare impose constraints on how work is done with meager understanding of the operational work at anything more than a superficial level and with little sensitivity to its operational demands. Unless that changes, the push for high-reliability organizing in healthcare will never be completely satisfied.

### User-centered design

User-Centered Design is offered by many as an intervention that will lead to the development of systems that will provide better clinical support [22]. It is questionable, however, whether a well-developed User-Centered Design process involves substantive design activity, where the term *design* refers specifically to those activities used to formulate the design solution as distinct from activities of analysis and assessment. Ellsworth, Dziadzko, O’Horo, et al. [23], although critical of the tendency within User-Centered Design to enter the development cycle late, nevertheless view User-Centered Design as directed at assessment of already-designed systems.

A systematic and comprehensive process of design proceeds through the stages of defining the problem, analyzing the work, generating solutions, and assessing prototypes and fielded systems; a nonlinear process that involves considerable iteration over adjacent and nonadjacent stages. LeRouge and Wickramasinghe [24] identified six stages for User-Centered Design (planning and feasibility, requirements, design, implementation, test and measure, and post-release) that correspond approximately to this process. Of fourteen activities identified for the design stage, only two suggest any form of design activity, the remainder being either analytic or assessment activities. Possibly, because of healthcare’s reliance on this limited view of design, clumsy and labor-intensive features such as data entry windows, drop-down menus, lists with check boxes, and alarms dominate as design features.

### Decision-centered design

Decision-Centered Design [25] emerged from research into Naturalistic Decision Making, the study of how people make decisions under time pressure and uncertainty [26]. The focus in Naturalistic Decision Making is on operational work that has meaningful consequences as undertaken by experienced (often expert) practitioners. Notably, decisions made in this context may have ambiguous or poorly-defined goals. Naturalistic deciding came to be viewed as a macro-cognitive process and the insights generated through its study prompted an extension of the naturalistic method of investigation to other macro-cognitive processes [26].

According to Crandall et al. [27], the term macrocognition refers to the collection of cognitive processes and functions that characterize how people think in natural settings. The designation as *macro* signifies that these cognitive processes relate directly to work goals. Cognitive processes such as situation assessment, diagnosing, deciding, planning, communicating, managing, directing, and collaborating are viewed as macro-cognitive versus, for example, micro-cognitive processes such as noticing, managing attention, accessing information, or assessing options. In this conceptual scheme, the micro-cognitive processes support the macro-cognitive processes when deliberately designed to do so.

Decision-Centered Design progresses through five phases; preparation (development of domain understanding for the research group), knowledge elicitation (identification of essential work-related cognition), analysis (isolation of leverage points for supporting work-related cognition), design (development of a design concept), and evaluation (impact estimate of the proposed design). It relies on context specific, incident-based narratives to isolate leverage points for supporting the macrocognition involved in challenging situations [25]. Decision-Centered Design identifies the key cognitive challenges and key elements of expertise involved in cognitive activity as a basis for generating design ideas that can support challenging cognitive work [28]. Design solutions may incorporate one or more of technological innovations, work process enhancements, or cognitive skills training [29].

### Cognitive modes

The creative design work undertaken within the framework of Decision-Centered Design is informed by views that align with Rasmussen’s problem-solving theory of cognitive modes [30]. A cognitive mode is a style of cognitive processing used to undertake cognitive work. Although Rasmussen does not refer to macrocognition as such [30, 31], the types of cognitive processes he refers to align with those identified as macro-cognitive by those who promote Decision-Centered Design [25–27].

Rasmussen offers three modes of cognition; skill-based, rule-based and knowledge-based [30, 31]. The skill-based mode has no conscious processing between perception and action, the rule-based mode is guided by sets of procedural instructions that specify sequences of actions, and the knowledge-based mode is grounded in conscious and explicit reasoning. Identification of the modes used in any cognitive work guides the design of supports for that work, although a cognitive modes analysis needs to identify not only modes in use but also the information that supports the work and the type of action to be taken (Table 1).

**Table 1:** Design rules for cognitive modes

Cognitive Mode	Information Support	Action Support
Skill-Based	Familiar perceptual patterns	Forms of direct manipulation
Rule-Based	Familiar perceptual patterns linked in procedural sequences with a consistent one-to-one mapping between the work constraints and the information provided at the interface	Manipulative capabilities linked directly to the perceptual forms that are intended to guide the action
Knowledge-Based	A knowledge resource, organized and indexed to support assembly of an adequate constellation of information for the current cognitive activity, in a form that allows its meaning in the current context to be readily evident, and filtered to exclude distracting elements	Knowledge bases, indexed and cross referenced. Succinct, targeted summaries of critical knowledge Planning and modelling tools

Cognitive work such as diagnosis might employ any one of the cognitive modes on their own or two or three in combination. A clinician who recognizes sepsis at a glance is working in the skill-based mode. One who consults a checklist is working in the rule-based mode. One who notices the signs but must consult a text book to resolve what they mean is working in the knowledge-based mode. It is also of value to consider why different workers use different modes for the same task. For this sepsis illustration, an experienced practitioner may prefer the skill-based mode, an inexperienced practitioner may prefer the rule-based mode, and a student may prefer the knowledge-based mode. A cognitive support must conform to the cognitive mode or modes preferred by those who will use the system. Watson and Sanderson [32], in their development of sonic displays for anesthesiology, have observed that the cognitive modes supported by the monitoring technology do not align well with those preferred by anesthesiologists.

### Workflow

Several of the papers we cite in our background review note that clinicians often express concern about the impact information technology has on their workflow (the series of activities necessary to complete a task). As will become evident in our subsequent discussion, clinicians establish workflow patterns in part to support macro-cognitive processing. For example, a clinician who is concerned with maintaining their situation awareness within a clinical setting is likely to develop a workflow that will help them access the information critical for sensemaking in the desired sequence and at the right time.

## Purpose

In this paper, we present an argument that the design of healthcare information systems must take account of the cognitive capabilities and skills deployed by all involved in patient care, including the patients themselves and their families [3]. In our review of the research into healthcare information systems as summarized above, we found no discussion of how cognitive processing could be disrupted by clumsy technological functions and no guidance in relation to dealing with those issues in design. For example, Karsh et al. [10] argue that the power of information technology should be focused on developing cognitive support that offers clinicians and patients assistance for thinking about and solving problems related to specific instances of healthcare. Karsh et al. [10] do not, however, suggest how to go about that. Greenhalgh et al. [4] argue that a sensemaking or soft-systems approach is rarely used prospectively in healthcare information systems design but offers no thought on how to implement such an approach.

We believe that the appropriate design methods are largely unknown and that these pleas to attend to cognitive issues will either be ignored or will turn designers towards a restricted view of User-Centered Design. We use this paper as an opportunity to introduce one cognitive design framework, Decision-Centered Design, that can address the concerns expressed by Karsh et al. [10] and Greenhalgh et al. [4]. It is one of several frameworks available within Cognitive Engineering [33].

Cognitive engineers focus on the design of technological support systems such as interfaces, information-entry systems and communication systems, and on human resource issues such as team design, organizational design, staffing, selection and training. In contrast to the technology-centric design assumptions of stable, routine and knowable work processes, cognitive engineers assume that healthcare work is demanding, fluid and unpredictable, being distributed and shared across a system of functionally interdependent actors and artefacts. Within the systems in which they are embedded, information technology artefacts are therefore ideally designed with respect to functional implications at the system level. The design goal for such an environment is to establish a robust system in which the human capability to perform cognitive work is optimized.

We use a case-study approach to emphasize cognitive issues as we develop our argument. Our first two case studies demonstrate the problems that accrue from taking a rational, exclusively rule-based approach to information system design while ignoring the cognitive subleties of healthcare work. Our second two case studies demonstrate the power of supporting cognition with a mix of skill-, rule-, and knowledge-based design strategies. Because we are offering an argument rather than a review or a survey, we deliberately selected case studies that illustrate the significance of cognition in healthcare and how that cognition might be supported with innovative design solutions that are not exclusively rule-based.

## The cognitive challenge

Decision-Centered Design evolved in response to the neglect by technology-centric design disciplines of the cognitive processes critical to the effective execution of human cognitive work. Here we illustrate the problems that emerge from designing cognitive support systems based on a technology-centric view of work practice by reference to two research papers that have assessed the efficacy of technological developments in healthcare.

### Case study: patient evacuation

An automated scheduling system was developed for the U.S. military to relieve healthcare staff of the task of scheduling patients for evacuation from first-point-of-care facilities [34]. For large-scale problems, the new system produced better schedules with less effort. However, the scheduling problem was dynamic in that staff could be confronted with an unscheduled evacuation request such as immediate transport of a seriously ill patient who required emergency medical treatment at a specified facility [35]. With diversion of an aircraft and crew to fill this urgent requirement, evacuation schedules for other patients could be disrupted so that the schedule would have to be adjusted.

Although common, schedule adjustment in response to dynamically unfolding needs had always been a challenge. However, in the process of manual scheduling, staff had implicitly developed an appreciation of potential resource options and conflicts, which they could use to adjust a schedule as consistent with new demands. In macro-cognitive terms, they had developed a useful level of situation awareness relating to available resources and potential conflicts via an implicit process of sensemaking. However, constraints imposed by the new automated scheduling system blocked staff from building that appreciation of potential options and conflicts so that staff were then ill-prepared to adjust schedules when needed.

Resource scheduling is a ubiquitous challenge in modern hospitals. For example, it can often be difficult in a large hospital to satisfy demands for in intensive care beds [36]. This is a problem that seems ideal for computerized support, but there is an ever-present concern that those who develop such a system will ignore many of the subtle cognitive processes that are critical to a satisfactory outcome.

### Case study: anesthesiology

A new, highly integrated, microprocessor-based physiological monitoring system for cardiac anesthesia was introduced into a cardiothoracic surgery unit to replace the functions of four single-sensor devices [37]. By centralizing the sensor data and the patient-monitoring functions in a single computer-based system, designers provided anesthesiologists with options for reorganizing windows on the screen and for viewing different representations of the same information. The most obvious interface difference from the previous assembly of discrete devices was the multi-layer menu structure that was activated via a touch screen.

Cardiac surgical patients are susceptible to rapid and profound hemodynamic changes, many of which can be life-threatening. However, the new system’s default numeric displays limited an anesthesiologist’s ability to assess the magnitude of rapid changes in blood pressure. This became an issue when the surgeon lifted the heart to feel the coronary blood vessels, an action that could cause blood pressure to fall rapidly. During such an event, the surgeon depends on the anesthesiologist to announce the correct blood pressures. These could be inferred readily from the default waveform representation of the old system, but the default numeric configuration of the new system encouraged a direct reading of numbers that changed too slowly to track blood pressure accurately. Although anesthesiologists learned to compensate by extrapolating the digital values, inexperienced residents sometimes failed to do so, which resulted in complaints from surgeons.

After considerable thought and experimentation, anesthesiologists developed a fixed-scale analog window that showed all blood pressures. Although it served the need when visible, this new window configuration had to be set up with a complex series of steps at the beginning of each case and, even then, was not entirely stable. An automatic window-management function could hide this new blood-pressures window as the anesthesiologist performed other tasks. That problem was largely resolved when an anesthesiologist discovered that the preferred screen configuration could be maintained by reserving screen space with modules that contained no useful information. Once this solution was known, the necessary window management could be completed during the low-workload period of system initialization.

Nevertheless, window management continued to be a problem when the anesthesiologist needed to measure cardiac output. Although cardiac output can be measured in 10 to 30 s, the result can be unreliable, and anesthesiologists often measure cardiac output two or three times in rapid succession to improve the estimate. With the new computer system, cardiac output was viewed on a special window brought to the screen by activating a screen label, but activation of this window had the side effect of removing the blood-pressures window, thereby degrading practitioner ability to detect rapid changes in blood pressure. This did not occur in the old system because the discrete devices displayed the data in parallel. Problematically, the time that measurement of cardiac output was most frequent coincided with the time that rapid changes in blood pressure were of most concern.

One consequence of providing multiple functions in a single device is that the control of these functions becomes more complicated. For example, a blood pressure channel was reset on the old system by pressing a physical switch on the front of a panel. With the new system, that channel reset required a series of screen activations. The most frequently used menu function of the computer system was measurement of cardiac output, a process requiring at least three menu activations. For the old, discrete device, that same function was activated with a single press of a mechanical button. Furthermore, errors were common. For example, an unintended menu activation could switch the system to a seldom-used region of the menu space. To recover, the anesthesiologist would typically escape back to the highest-level of the menu and then navigate through the menu tree back to the desired location. Although workable, this sort of disruption was clearly undesirable.

Evident in this illustration is that much of an anesthesiologist’s workflow is organized to support sensemaking. Workflows established for that purpose in the new system, which revolved largely around window management, contained many more steps and were more fragile than in the old system. Sensemaking was disrupted when displays either obscured information or provided it in formats that could not be interpreted at a glance. In addition, there was a flow-through to the macro-cognitive process of collaboration between anesthesiologist and surgeon, which was jeopardized when the anesthesiologist found it difficult to transform display readings as required by the surgeon.

### Summarizing the cognitive challenge

The problems experienced in use of the systems described in our first two case studies [34, 37] resulted primarily from the failure to adopt a cognitively-relevant systems approach in design and implementation. A techno-centric mindset took the benefits of computerization for granted, directing attention away from the cognitive demands of healthcare work. Obvious functionality was assigned by default and subtle but critical functionality ignored.

More generally, these design projects appear to have been dominated by a technological imperative with no apparent concern for the cognitive challenges posed by the work.

- The developers of the automated scheduling system were unaware of the need for evacuation schedulers to build awareness of potential resources and conflicts. After all, if the scheduling system had worked as intended, the requirement for any human cognitive work would have been minimal.
- The developers of the microprocessor-based physiological monitoring system for cardiac anesthesia appear to have understood the work well enough to know what data and what functions had to be provided, which could have been understood by an in-depth examination of the technology to be replaced, but they showed no understanding of the strategies or workflows used by anesthesiologists.

These were failures of neglect, ones that could have been avoided with the right sort of analysis and design expertise. The need to design computerized systems so that they do not disrupt cognitive work and do not add onerous cognitive tasks may be challenging, but the design efforts behind the two systems of our case studies show no evidence that this challenge was even recognized.

## Cognitive engineering

Cognitive engineers can establish a bridge between the expertise and needs of those who are involved in patient care and those who have the technical expertise to build a computerized information system. In establishing this bridge, cognitive engineers resolve two issues, one relating to analysis of the cognitive work and the other related to design of a support system for the cognitive work [e.g., 32].

### Analysis of cognitive work

The analysis of cognitive work explores the way workers pursue work goals and resolve cognitive challenges of that work, often in novel, subtle, and creative ways, and it seeks to uncover the strategies and tactics employed by experts as they engage in non-routine or challenging incidents [28]. The analysis of cognitive work requires structured methods because, on the one hand, healthcare professionals have expertise in their own specialty, but they cannot always articulate the subtle aspects of their expertise as it applies to their work. Nor can they frame even the knowledge they can articulate in terms that software engineers and information technology specialists can translate into design specifications. Finally, even knowledgeable healthcare professionals have only limited understanding of healthcare complexities beyond the domain of their own expertise. The design of a healthcare support system requires considerable specificity but inevitably, healthcare professionals will be able to make only general recommendations outside their own specialty.

On the other hand, software engineers and information technologists who build healthcare systems have only a limited appreciation of the demands and complexities of healthcare work. They are possibly induced to believe they know enough about healthcare because they do interact with the healthcare system as patients. While an understanding of *work-as-imagined* is useful, cognitive design requires a deeper analysis of *work-as-done*. If information technologists think at all about cognition, they will more likely be guided by common fallacies [38] than by scientifically sound principles.

### Design of cognitive support

Our first two case studies [34, 37] illustrate how an in-depth, cognitively oriented work analysis can uncover the cognitive complexity and cognitive subtlety of the work undertaken by healthcare practitioners, and they illustrate the character of the cognitive issues that designers of information technology support systems need to address. However, neither of those studies proceeded to the step of designing a solution for the cognitive issues they identified.

Tufte [39] observes that to be effective as decision support tools, information displays need to reflect the cognitive structure of the problem. Among his numerous illustrations of this principle, Tufte refers to an innovation by Dr. John Snow who identified the source of a cholera epidemic in London in 1854 by visually mapping cases against location to reveal that cases were clustered around one fresh water pump. Snow brought the epidemic under control by removing the handle from that pump. Notably, Snow’s cognitively-oriented solution to this problem was technologically simple, low-cost and remarkably effective. This principle, that displays should reflect the cognitive nature of the problem, can be restated as a call to represent the affordances [32, 40, 41] or the functionality [42] of the work and is one of the central design goals of cognitive engineering.

Powsner and Tufte [43] were guided by this cognitive principle in their redesign of the daily hospital record typically located at the end of a patient’s bed. Powsner and Tufte argued that the traditional form of the hospital record serves well as an archive but is not organized in a way that supports problem solving for patient care. They developed a detailed, one-page graphical representation that showed the time course of important physiological parameters, symptoms, and treatments in an array of small, high-resolution graphs with identical formats. Distinctive labeling allowed healthcare practitioners to converge readily on the information they were seeking on any occasion; for example, a single piece of information such as when the patient had commenced treatment with a specific drug, or a cluster of information such as the progression of a group of symptoms over days or weeks. The result was a summary picture of patient status that enabled the healthcare practitioner to examine relations between findings and treatments and to assess alternative diagnostic and management strategies. This work by Powsner and Tufte illustrates how it is possible to design a cognitive support tool that goes beyond the standard suite of design options of window hierarchies, drop down menus, text boxes and check lists or the common rule-based strategies that rely on written procedures, alarms and algorithms for clinical decision support.

## Facing the challenge

The design work undertaken by Powsner and Tufte was, however, directed at a relatively simple and well-understood work problem and did not employ any in-depth analysis of cognitive issues. Cognitive engineering is aimed at developing innovative cognitive support solutions for work that has subtle and hidden complexities. That requires both comprehensive analysis and creative design.

In the following analysis, we focus on projects that used Decision-Centered Design because it is a mature framework that is widely used outside of healthcare and is comparatively easy to understand. Decision-Centered Designer deviates from an alternative cognitive engineering strategy of comprehensive analysis [42] by focusing on leverage points, those challenging work activities that offer opportunities for substantive performance enhancement if addressed with innovative cognitive design solutions.

### Case study: colorectal cancer screening

Although managing and tracking colorectal cancer screening appears to be a simple problem readily resolved with rule-based reminders, screening rates remain well short of the 80% target established by The National Colorectal Cancer Roundtable [44]. Militello, et al. [45] developed a colorectal Screening and Surveillance App that was designed to work with the US Veterans Health Administration’s Computerized Patient Record System. For their development, they used Decision-Centered Design to identify the challenging decisions faced by clinicians in managing and tracking colorectal cancer screening and then design a cognitive support system for that work.

The development progressed through four design iterations. Each design iteration was different but, as required by the five-phase Decision-Centered Design strategy, each involved reviews of documents, discussions with or observations of healthcare practitioners, identification of leverage points, development of a design concept, and evaluation. This cognitively-focused strategy identified three important macro-cognitive processes that were troublesome within the current system and the micro-cognitive processes that posed challenges to healthcare professionals attempting to execute those macro-cognitive processes.

Sensemaking was troublesome because it was difficult to locate and integrate the information needed to construct a useful narrative about the patient. Details of a patient’s colorectal cancer screening history were stored in disparate places, again demonstrating how electronic information systems can be so poorly configured that they impede rather than facilitate the work.

Problem detection was troublesome because the electronic health record did not help healthcare professionals notice anomalies that might require non-routine action. Sometimes, the electronic record would indicate erroneously that the patient was due for screening. Because the clinical reminder provided no rationale for the recommendation, the primary-care provider would typically order the test, which would then be rejected by the testing clinic for no reason apparent to the primary-care provider. Nor did the electronic record clearly show what happened after a test was ordered. Primary-care providers found it difficult to ascertain whether support staff and patients followed up as needed to complete the order.

A third macro-cognitive challenge involved collaboration between primary-care providers and patients. Screening rates are affected by patient attitudes and it is incumbent on primary-care providers to engage effectively with patients to help them reach an informed decision. Patients will typically proceed with screening only if they are aware that it is beneficial and accessible. Providers reported that patient education in relation to colorectal cancer screening is time consuming; a challenge that is exacerbated when the primary-care provider is pressed for time and the patient has limited communication skills.

Militello, et al. [45] configured their colorectal Screening and Surveillance App to support these three macro-cognitive processes. The App encouraged a mix of skill-, rule-, and knowledge-based processing by use of an information representation that helped the primary-care provider navigate skillfully to key information that would support knowledge-based processing in service of complex macro-cognitive processes such as sensemaking, problem detection, and collaborative decision making. Rule-based processing was supported by a salient display of reminders.

Militello, et al. [45] also developed a one-page educational brochure designed to help a primary-care provider discuss screening options for, and common misconceptions about colorectal cancer with their patients. Although the Center for Disease Control already had a two-page brochure on this topic, this new one-page brochure was designed to be easier to view on a screen, easier to print, and less daunting to read [46].

The evaluation phase in the fourth design iteration showed that, given the support of the prototype Colorectal Screening and Surveillance App, primary-care providers answered questions about patients more accurately and found relevant information more quickly compared to those using only the Computerized Patient Record System [45]. Primary-care providers also reported reduced mental effort, assessed subjectively on a nine-point scale from extreme to none [47], and rated the Screening and Surveillance App positively for usability [45]. A separate evaluation showed that the one-page educational brochure improved knowledge of and openness to colorectal cancer screening to a degree equivalent to that achieved with the more cumbersome and detailed two-page brochure from the Center for Disease Control [46].

### Case study: self-management of type II diabetes

Klein and Lippa [48] studied the challenges posed by patient self-management of Type II diabetes. They interviewed diabetes patients to explore how well they understood their disorder and its management demands. The interviews identified the explicit knowledge, macro-cognitive skills, and mental models used to structure glucose self-management activities and decisions. To supplement the results of their interviews, Klein and Lippa reviewed relevant documents and websites, surveyed educational interventions, and observed diabetes management training classes. They also reviewed discussions in an American Diabetes Association chat room. By use of these methods, they built up a comprehensive picture of the macrocognition that impacted the effectiveness of patient self-management for Type II diabetes.

Their analyses of this information indicated that the explicit knowledge provided to patients from many sources could be useful, but the complexity of diabetes self-management could be overwhelming. Many patients found it difficult to deal with changes in routine that accompany events such as illness, stress, and travel. Even people with considerable explicit knowledge encountered challenges because that knowledge was not linked effectively to the situational constraints imposed by non-routine events. For example, a diet that worked well could be disrupted by the social expectations that accompany holidays or other communal interactions.

From analysis of their data, Klein and Lippa [48] concluded that education for diabetes self-management emphasizes rules and procedures that are overly complex and that do not respond well to the dynamic challenges that patients face in managing diabetes. They proposed that glucose-level management is analogous to the regulation of a complex, dynamic system (see Table 2 for the distinction between rule-based and dynamic control). Although, Klein and Lippa [48] acknowledge the value of rules and procedures, they argue that no rule-set can be adequate and that patients need an instructional program that can sensitize them to the situated knowledge associated with non-routine events, and can help them build skill with the macro-cognitive processes that support dynamic control [48]. Subsequently, they established that patients who relied on dynamic control (detecting vital information, responding to feedback, anticipating perceived trends) rather than rules are better able to maintain healthy blood glucose levels [49].

**Table 2:** Rule-based versus dynamic control

An egg is boiled by reference to a rule; the time known to achieve the desired set. However, this rule is context–dependent; a meaningful change in altitude requires an adjustment in cooking time. In contrast, an egg is fried by use of dynamic control. The cook monitors several sources of information, possibly adjusting the heat to speed or slow the process, and may even generate useful information by shaking the pan. Dynamic control is robust in the face of changes in context.	

## Conclusions

Healthcare work is, to a considerable extent, cognitive. Our first two case studies [34, 37] illustrate the problems that emerge absent any serious and comprehensive attempt to understand the cognitive processes involved in healthcare. The design of supporting technology must be sensitive to the cognitive demands of the work and to the informal cognitive strategies employed by those play a meaningful role in the care of a patient. Most critically, we should not mandate procedures, or field technologies that block the progress towards competency, or that force those who are performing effectively to modify or abandon the cognitive processes on which their competency is based. Our final two case studies [45, 48] illustrate how established analysis and design tools from Decision-Centered Design can be deployed to develop effective cognitive support.

There are, however, forces working against widespread application of a systematic approach to cognitive design. Healthcare already has strategies for technology acquisition. They generally involve a select committee of hospital staff members with considerable experience in administrative or clinical matters [9, 13]. Although that select committee may be tasked to identify the system best suited to meet their hospital’s needs from those already available on the market [9], its members are unlikely to have any appreciable expertise in device evaluation [13]. That committee may subsequently develop familiarization and training programs for the new system but is unlikely to have any other role [9]. As members of a select committee, one of us (GL) has personally been involved in an alternate strategy of developing a requirements list, which was then passed administratively to a technology development team. Neither approach is likely to result in a system that takes account of the cognition and workflows of those involved in patient care.

When the resulting system turns out to be less than effective, and sometimes unconscionably clumsy, the fault is rarely attributed directly to the technology acquisition process. Even more rarely is the disappointment translated into a general lesson for technology development in healthcare. The failure is typically assessed from a parochial and narrow viewpoint without consideration of the depth and complexity of the design challenge [8]. Furthermore, the successes that emerge from a comprehensive and balanced cognitive analysis and design effort are not widely appreciated. We hope that this article will serve to expand appreciation of what is possible.

What should be clear from our review of the positive case studies is that cognitive analysis and cognitive design are demanding in terms of time and effort. The effort is not, however, excessive in relation to that required for the technology development. Indeed, reflection on success stories within cognitive engineering [50, 51] suggest that the costs associated with an organized effort directed at cognitive analysis and cognitive design are modest in relation to costs of technology development. Furthermore, those success stories indicate that the products of cognitive design can result in performance gains that exceed by orders of magnitude the performance gains possible with technology-focused solutions and could thereby help healthcare achieve much-desired gains in efficiency, productivity and safety.
